# Bioavailability Enhancement of Cepharanthine via Pulmonary Administration in Rats and Its Therapeutic Potential for Pulmonary Fibrosis Associated with COVID-19 Infection

**DOI:** 10.3390/molecules27092745

**Published:** 2022-04-24

**Authors:** Jian Li, Guangrui Chen, Zhiyun Meng, Zhuona Wu, Hui Gan, Xiaoxia Zhu, Peng Han, Taoyun Liu, Fanjun Wang, Ruolan Gu, Guifang Dou

**Affiliations:** 1 Department of Pharmaceutical Sciences, Beijing Institute of Radiation Medicine, Beijing 100850, China; ruirui15855751020@163.com (G.C.); mengzhiyun@vip.163.com (Z.M.); wznphd@126.com (Z.W.); ganh2003@163.com (H.G.); 13681022512@163.com (X.Z.); 15901135949@163.com (P.H.); liutaoyun@163.com (T.L.); wfjjjup@163.com (F.W.); dougf@bmi.ac.cn (G.D.); 2 School of Basic Medical Sciences, Anhui Medical University, Hefei 230032, China

**Keywords:** cepharanthine, COVID-19, pulmonary delivery, anti-fibrosis, UPLC-MS/MS, bioavailability

## Abstract

Cepharanthine (CEP) has excellent anti-SARS-CoV-2 properties, indicating its favorable potential for COVID-19 treatment. However, its application is challenged by its poor dissolubility and oral bioavailability. The present study aimed to improve the bioavailability of CEP by optimizing its solubility and through a pulmonary delivery method, which improved its bioavailability by five times when compared to that through the oral delivery method (68.07% vs. 13.15%). An ultra-performance liquid chromatography tandem-mass spectrometry (UPLC-MS/MS) method for quantification of CEP in rat plasma was developed and validated to support the bioavailability and pharmacokinetic studies. In addition, pulmonary fibrosis was recognized as a sequela of COVID-19 infection, warranting further evaluation of the therapeutic potential of CEP on a rat lung fibrosis model. The antifibrotic effect was assessed by analysis of lung index and histopathological examination, detection of transforming growth factor (TGF)-β1, interleukin-6 (IL-6), α-smooth muscle actin (α-SMA), and hydroxyproline level in serum or lung tissues. Our data demonstrated that CEP could significantly alleviate bleomycin (BLM)-induced collagen accumulation and inflammation, thereby exerting protective effects against pulmonary fibrosis. Our results provide evidence supporting the hypothesis that pulmonary delivery CEP may be a promising therapy for pulmonary fibrosis associated with COVID-19 infection.

## 1. Introduction

The ongoing global pandemic coronavirus disease 2019 (COVID-19), caused by severe acute respiratory syndrome coronavirus 2 (SARS-CoV-2), has attracted great attention owing to its rapid transmission, high mortality rate, and the lack of a specific treatment regime [1,2]. Moreover, the emergence of new mutant strains of SARS-CoV-2 has further complicated the epidemic, making it difficult to control [3,4,5]. Despite the successful developments and applications of COVID-19 vaccines [5,6,7], new outbreaks continue to be reported by several countries and regions [8,9]. Therefore, the application of novel and effective therapies along with vaccines is urgently needed [10]. As a reliable resource against diseases for millennia, herbal medicines and natural antivirals with the least toxicity may be a good choice against SARS-CoV-2 [5].

**Figure 1:**
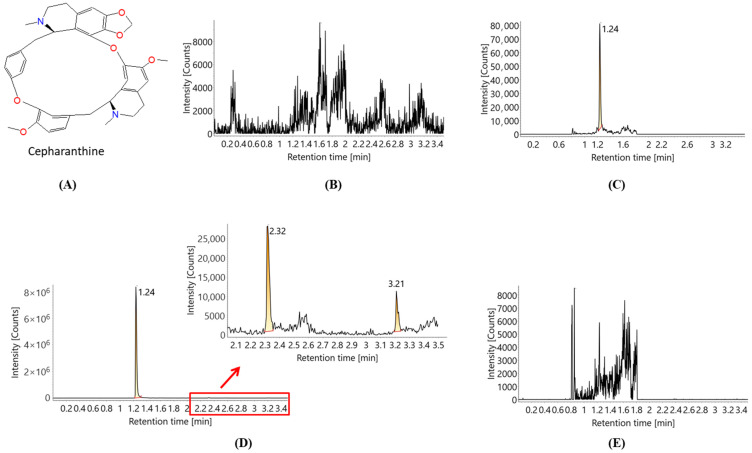
Chemical structure and MRM chromatograms of CEP in rat plasma samples: (**A**) chemical structure of CEP; (**B**) blank sample; (**C**) standard sample at the LLOQ (0.5 ng/mL); (**D**) top calibration standard (100 ng/mL) by multiple gradient elution; (**E**) blank sample after injection of the top calibration standard.

Cepharanthine (CEP), which is isolated from the plant *Stephania cephalantha* Hayata, is a natural bisbenzylisoquinoline alkaloid possessing an elliptic macrocyclic structure (Figure 1A) [11]. CEP has been reported to possess anti-oxidative, anti-inflammatory, anti-parasitic, immunomodulating, and antiviral properties [12,13,14], indicating its potential application in the treatment of viral diseases, such as COVID-19. In particular, CEP has been confirmed to exert inhibitory effects on coronavirus, including SARS-CoV and HCoV-OC43 [14,15,16]. Most importantly, recent studies have found that CEP could significantly impair the infectivity of SARS-CoV-2 and its subsequent replication in vitro [10,17,18,19,20]. Therefore, CEP seems like a promising candidate for anti-COVID-19 therapeutics [9,10,15]. However, the drug development and application of CEP are evidently restricted owing to its poor solubility and low bioavailability [21]. After its oral administration, CEP is eliminated very quickly, making it difficult to maintain a high level of CEP in the blood [22]. For COVID-19 patients, the lung is one of the main organs and lesions that the virus attacks [23,24]. Considering the huge surface area of alveoli and the abundant capillaries in the lungs, pulmonary delivery of the drug can promote its absorption, reduce the first-pass effect, and effectively improve bioavailability, which is considered the most potent therapy for pulmonary diseases [25,26,27].

Moreover, clinical and radiographic reports have indicated that most COVID-19 patients, especially severely affected ones, present with ground-glass opacities, consolidation in different degrees, bronchial vascular bundle thickening, and interstitial changes in the lungs [28,29,30]. Similar symptoms were also reported in past pandemics of severe acute respiratory syndrome (SARS) and Middle East respiratory syndrome (MERS) [24]. Pulmonary fibrosis is associated with permanent pulmonary architectural distortion and irreversible lung dysfunction, which may affect the patients’ quality of life and survival [28]. Therefore, pulmonary fibrosis may be one of the major complications and sequela in COVID-19 patients [31]. However, the pathogenesis of pulmonary fibrosis remains unclear, with only a few reliable antifibrotic agents reported for COVID-19 currently [32,33]. As the epidemic continues, pulmonary fibrosis has recently attracted more and more attention and follow-up studies. Among them, a lung fibrosis model induced by bleomycin (BLM) was recommended and used widely because this model might actually be beneficial in COVID-19, both in the acute phase of the disease and in preventing long-term complications [34,35,36,37,38,39].

The present study aimed to improve the bioavailability of CEP and evaluate its therapeutic efficacy on anti-lung fibrosis in rats. First, the problem of poor solubility was solved, and the CEP solution was subsequently available for pulmonary administration. Then, a bioanalytical method for the determination of CEP was established, validated, and then successfully implemented for the subsequent investigation of pharmacokinetics and bioavailability. Finally, the anti-pulmonary fibrosis potential of CEP was evaluated in a rat model through the pulmonary delivery mode.

## 2. Results

### 2.1. UPLC-MS/MS Method Development and Validation

A simple and rapid bioanalytical method was developed based on the ultra-performance liquid chromatography tandem-mass spectrometry (UPLC-MS/MS) system to detect the concentration of CEP in the rat plasma. Different instruments and varying conditions were tested to optimize the settings of chromatography and MS. Initially, a carryover was recorded in different LC-MS/MS systems, which was serious and exceeded 25% of the lower limit of quantification (LLOQ). After a series of chromatographic optimizations, we noted that elution with multiple gradients played a key role in eliminating the carryover, especially on the analytical column. As shown in Figure 1D, the retention time of CEP is 1.24 min, and two obvious eluting peaks (2.32 min and 3.21 min) appeared by using the multiple gradient elution, which ensures CEP was fully eluted from the column. Quantitation was performed using the optimized MRM transitions of *m*/*z* 607.2 → 174.1 for CEP and *m*/*z* 237.1 → 194.0 for internal standard (IS, carbamazepine) in a positive mode.

To demonstrate the specificity of the assay, six different batches of blank rat plasma were tested, and no endogenous interference was observed at the retention time as the analyte and IS. The calibration curve of CEP was linear from 0.5 to 100 ng/mL with a regression coefficient (r^2^) > 0.997. The intra- and inter-assay precision and accuracy are summarized in Table 1. These results demonstrate that the precision and accuracy values were well within the acceptable range of 15%. As shown in Table 2, the extraction recovery of CEP ranged from 93.9% to 94.9% at three quality control (QC) concentration levels and the matrix effect was in the range of 98.9–105.8% with the coefficient of variation (CV%) < 15%, which confirmed that the endogenous matrix would not affect the quantitative analysis. For stability evaluation, the calculated RE (%) ranged from −1.74% to 10.28% with CV% < 15%, indicating that CEP in the rat plasma and the stock solutions was stable under bench-top, autosampler, short- and long-term storage conditions, and freeze/thaw conditions. Finally, the detection accuracy and precision for dilution integrity of 1:100 dilution were 8.51% and 1.29%, respectively (both within ±15%), which confirmed that samples above the upper limit of quantitation could be correctly quantified after 100-fold dilution.

### 2.2. Pharmacokinetics and Bioavailability Study

**Figure 2:**
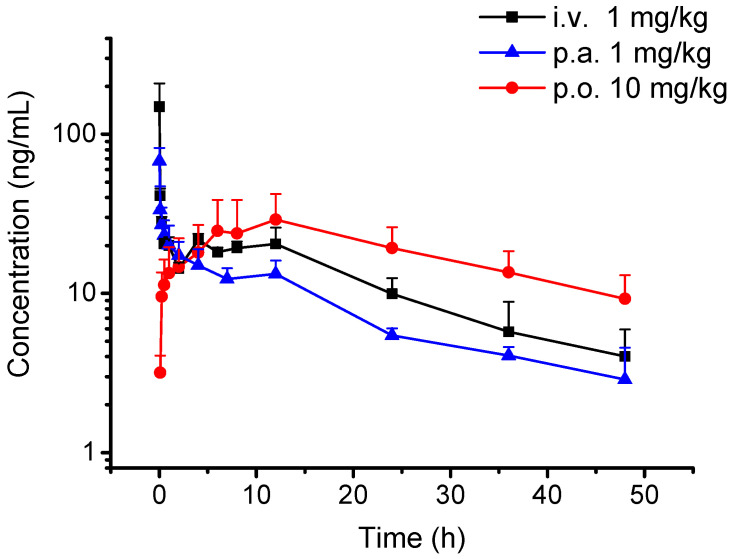
Mean plasma concentration-time profiles of CEP after intravenous (i.v. 1 mg/kg), oral (p.o. 10 mg/kg), and pulmonary administration (p.a. 1 mg/kg) (*n* = 4).

The validation method was applied to characterize the PK properties of CEP in rats. The mean plasma concentration-time profiles of CEP after intravenous, oral, or pulmonary administration are presented in Figure 2. The main pharmacokinetic parameters were calculated and are summarized in Table 3. After 1 mg/kg intravenous or pulmonary administration, CEP reached its maximum concentration (C_max_) in the plasma immediately at the first sampling time (1 min) with C_max_ values of 148.8 ± 60.1 ng/mL and 65.3 ± 16.1 ng/mL, elimination half-life (t_1/2_) of 2.80 ± 0.42 h and 16.35 ± 1.67 h, and areas under the curve (AUC_(0-t)_) of 576.2 ± 114.1 h·ng/mL and 392.2 ± 43.7 h·ng/mL, respectively. After oral administration of 10 mg/kg, the C_max_, T_max_, t_1/2_, and AUC_(0-t)_ were 31.8 ± 14.6 ng/mL, 13.50 ± 7.55 h, 17.15 ± 3.14 h, and 757.8 ± 144.7 h·ng/mL, respectively. The CEP bioavailability (F) of pulmonary administration was proven to be 68.07%, which improved over 5-fold when compared with that of oral administration (F, 13.15%).

### 2.3. In Vivo Anti-Fibrotic Effect

#### 2.3.1. The Effect of CEP on the Lung Index

**Figure 3:**
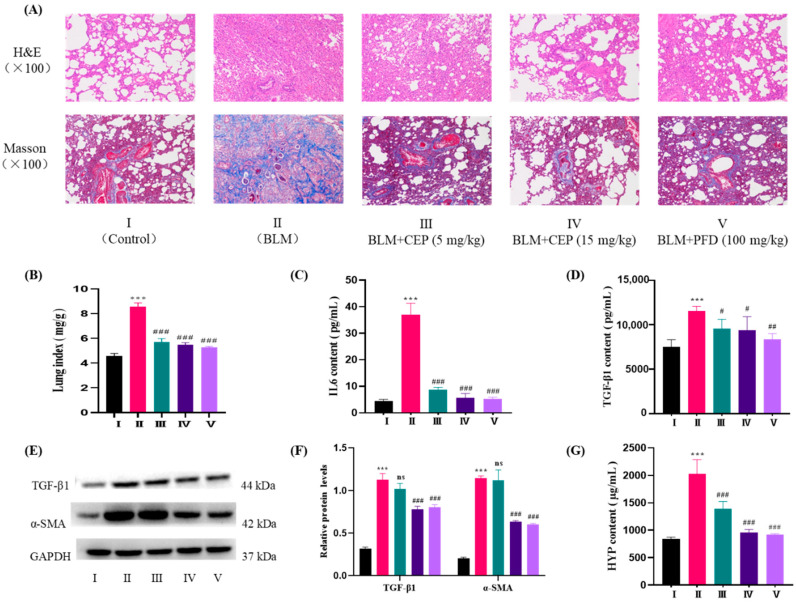
CEP ameliorates BLM-induced pulmonary fibrosis in rats. (**A**) representative H&E and Masson staining of lung tissues from each group; (**B**) the lung index; (**C**) IL-6 level in serum; (**D**) TGF-β1 level in serum; (**E**,**F**) protein expression of TGF-β1 and α-SMA in lung tissue; (**G**) hydroxyproline content in lung tissue. I: Control group, received normal saline by pulmonary administration for 21 consecutive days (once a day); II: BLM-induced pulmonary fibrosis group, treated with BLM (5 mg/kg) by pulmonary administration on the first day and received normal saline for 20 consecutive days (once a day); III: BLM + CEP 5 mg/kg group, treated with BLM (5 mg/kg) on the first day and received CEP by pulmonary administration for 20 consecutive days (5 mg/kg, once a day); IV: BLM + CEP 15 mg/kg group, treated with BLM (5 mg/kg) on the first day and received CEP by pulmonary administration for 20 consecutive days (15 mg/kg, once a day); V: BLM + PFD 100 mg/kg group treated with BLM (5 mg/kg) on the first day and received PFD by oral administration for 20 consecutive days (100 mg/kg, once a day). Data are presented as the means ± SD (*n* = 6). *** *p* < 0.001 versus control group. # *p* < 0.05, ## *p* < 0.01, ### *p* < 0.001 and ns (no statistical difference) versus BLM group.

All animals from each group survived until the end of the experiment. Anesthesia and daily treatment (Table 4) were well-tolerated by the experimental rats. On the last day, the lung index of the rats in the BLM group increased significantly in comparison with that in the control group (8.55 ± 0.32 mg/g vs. 4.58 ± 0.19 mg/g, *p* < 0.001; Figure 3B). Meanwhile, the treatment with CEP or pirfenidone (PFD) revealed an obvious lowering effect on the lung index (5.27–5.71 mg/g) when compared with that of the BLM group (*p* < 0.001).

#### 2.3.2. The Effect of CEP on the Histopathological Findings

As illustrated in Figure 3A, hematoxylin and eosin (H&E) staining revealed a normal alveoli structure of the control group without any obvious shedding of the epithelial cells in the bronchial cavity and inflammatory changes. The BLM group lung sections showed that the pulmonary architecture was significantly destroyed, including collapsed alveolar spaces, alveolar septal thickening, and heavy infiltration of the inflammatory cells. A distinct feature of pulmonary fibrosis is excessive collagen deposition in the lungs. In addition, Masson staining revealed BLM-increased collagen fiber hyperplasia in the interstitial lung spaces with the nearly complete destruction of the alveolar architecture. On the other hand, treatment with CEP or PFD could ameliorate the destruction, and inflammation and collagen accumulation were prominently reduced.

#### 2.3.3. The Effect of CEP on IL-6 and TGF-β1 Contents in the Serum

The contents of cytokines IL-6 and TGF-β1 were detected by enzyme-linked immunosorbent assay (ELISA). The rats in the BLM-treated group demonstrated a higher level of IL-6 (4.45 ± 0.59 pg/mL) and TGF-β1 (11538.9 ± 531.7 pg/mL) in the serum relative to that of the control group (IL-6, 37.09 ± 4.28 pg/mL; TGF-β1, 7492.9 ± 821.6 pg/mL, *p* < 0.001). Both CEP and PFD significantly decreased the levels of IL-6 (5.35–8.66 ng/mL) and TGF-β1 (8372.1–9572.2 pg/mL) contents in the serum when compared to that in the BLM-treated group (Figure 3C,D).

#### 2.3.4. Effect of CEP on TGF-β1 and α-SMA Protein Expressions in the Lung Tissues

As the key markers of fibroblast activation and differentiation [40], the TGF-β1 and α-SMA protein expression were analyzed by Western blotting (Figure 3E,F). The data demonstrated that BLM produced a marked elevation in the protein expression levels of TGF-β1 and α-SMA relative to those in the control group (*p* < 0.01). However, treatment with CEP (15 mg/kg) or PFD significantly downregulated the TGF-β1 and α-SMA protein levels in the lung tissues when compared with those in the BLM group (*p* < 0.01). CEP at a dose of 5 mg/kg displayed no significant effect.

#### 2.3.5. The Effect of CEP on Hydroxyproline (HYP) Content in the Lung Tissues

The content of HYP in the lungs was determined as a quantitative measure of collagen deposition [41]. As shown in Figure 3G, BLM considerably increased the HYP content in the lung tissues (2033.2 ± 253.8 μg/mL) when compared to that in the non-treated group (841.4 ± 32.3 μg/mL, *p* < 0.001). Notably, the rats receiving CEP or PFD exhibited a marked reduced effect on the content of HYP in comparison with that in the BLM group. Specifically, at a dose of 15 mg/kg, CEP displayed a better effect in reducing the HYP content than that of 5 mg/kg, suggesting that its effect may be dose-dependent.

## 3. Discussion

Pulmonary fibrosis is a chronic, progressive, and irreversible disorder in interstitial lung diseases [42]. Past reports have indicated that SARS-CoV-2, using an angiotensin-2-converting enzyme (ACE2) as a cell receptor for transmission, can induce interstitial lung damage at first and then parenchymal lesions [43]. The injury of the alveolar epithelium could induce the proliferation and differentiation of fibroblasts into myofibroblasts, excessive deposition of collagen, and gradual destruction of lung architecture [44]. Presently, pulmonary fibrosis is a recognized sequela of SARS-CoV-2 infection, which has attracted significant attention and follow-up research [23,24].

To explore the potential role of CEP in pulmonary fibrosis, a rat model was successfully established by BLM treatment. The BLM-induced model was preferred for this study, owing to its ease of induction, a short period of induction, and good reproducibility but also because of its applicability to COVID-19, both in the acute phase of the disease and in preventing long-term complications [34]. The model was recommended and extensively applied in the research of pulmonary fibrosis associated with COVID-19 infection [35,36,37,38,39]. For example, remdesivir [37] and Qingfei Paidu decoction [38], which are the main clinically recommended treatments for COVID-19 patients, were evaluated as an anti-fibrosis agent by using a BLM-induced model. Initially, we adopted the conventional method by a single intratracheal instillation of BLM through a surgical pre-incision on the neck [39]. However, we found that the distribution of fibrosis in some models was uneven, with nodules in the lungs despite sufficient repetitive shaking, and an incision on the neck was detrimental to consecutive daily dosing and the health of the rat, subsequently. To avoid these issues, we developed a new route of pulmonary administration for BLM via intratracheal nebulization using a nebulizer and a laryngoscope, which did not require incision surgery. In addition, we optimized the anesthesia method and selected isoflurane inhalation instead of intraperitoneal injection of pentobarbital sodium. The experimental rats anesthetized with isoflurane showed the advantage of shorter recovery time, better adaptability to pulmonary drug delivery, and higher survival rate than that with pentobarbital sodium.

Previous studies showed that CEP had multiple pharmacological activities including immunomodulating, anti-oxidative, anti-inflammatory, and anti-coronavirus, especially [12,13,14,15,16]. In addition, accumulating evidence demonstrates that CEP may be a potential agent against SARS-CoV-2 infection. Since the outbreak of COVID-19, CEP was first reported to exhibit antiviral effects for pangolin coronavirus GX_P2V, a SARS-CoV-2-related coronavirus [45]. Next, a combined treatment of CEP and nelfinavir was verified to be highly effective in a SARS-CoV-2 cell culture model [10]. Subsequently, some researchers further confirmed the antiviral effect and the possible mechanism of CEP against SARS-CoV-2 [17,18,19,20]; one of these studies reported that CEP demonstrated the best efficacy of anti-SARS-CoV-2 with an EC50 of 0.1 μM after screening 1900 clinical safe drugs [20]. However, there is no report on CEP in the treatment of pulmonary fibrosis associated with COVID-19 infection. In this research, we confirmed that CEP could significantly decrease the BLM-induced alveolar inflammation level, the degree of collagen deposition, lung index, and the levels of TGF- β1, α-SMA, IL-6, and HYP in the serum or lung tissues. All the above evidence suggests that CEP has comprehensive therapeutic potential for COVID-19 infection. In addition, we improved the solubility of CEP in water, and its bioavailability through pulmonary administration was tested to be 68.07%, increasing over 5-fold when compared with that through oral administration (F, 13.15%). Moreover, CEP can be directly delivered to the lung lesions via pulmonary administration, thereby exerting its therapeutic effects rapidly. These results indicate that CEP through pulmonary delivery may be a potential therapy for lung fibrosis and may provide a further reference for the treatment of COVID-19 patients.

## 4. Materials and Methods

### 4.1. Reagents and Materials

CEP (purity 99.7%) was purchased from Topscience Co., Ltd., Shanghai, China. Carbamazepine (purity 100%, National Institutes for Food and Drug Control, Beijing, China), BLM (purity 98.81%, MCE, Dallas, TX, USA), pirfenidone (purity 98%, Shanghai Macklin Biochemical Co., Ltd., Shanghai, China), and isoflurane (Shanghai Yuyan Scientific Instrument Co., Ltd., Shanghai, China) were obtained. LC/MS grade methanol and formic acid were purchased from Fisher Scientific (Fair Lawn, NJ, USA).

### 4.2. Experimental Animals

Male Sprague Dawley (SD) rats weighing 200 ± 20 g were purchased from Beijing Vital River Laboratory Animal Technology Co., Ltd. (license SCXK (Beijing) 2016-0011). Feeding was performed under specific pathogen-free conditions for 1 week before the experiments. All animal experiments were approved and supervised by the Institution of Animal Care and Use Committee, Academy of Military Medical Science (IACUC-AMMS, Beijing, China).

### 4.3. UPLC-MS/MS Method Development

#### 4.3.1. Chromatographic and Mass Spectrometry Conditions

The bioanalytical method for quantifying CEP was performed on the Acquity UPLC/Xevo TQ-S Triple Quadrupole MS (Waters, Milford, MA, USA). The samples were separated on the Waters BEH C18 column (2.1 × 50 mm, 1.7 μm) at 45 °C. A gradient elution program was conducted with the mobile phase A (0.1% formic acid in methanol) and mobile phase B (0.1% formic acid in water) as follows: 0–0.5 min, 10% A; 0.5–1.0 min, 10–90% A; 1.0–1.5 min, 90% A; 1.5–1.7 min, 90–10% A; 1.7–1.9 min, 10–90% A; 1.9–2.1 min, 90% A; 2.1–2.3 min, 90–10% A; 2.3–2.5 min, 10–90% A; 2.5–2.7 min, 90% A; 2.7–4.0 min, 10% A. The flow rate was set to 0.4 mL/min for an injection volume of 5 μL. MS analysis was performed using the positive multiple reaction monitoring (MRM) mode with an electrospray ionization (ESI) source. The MRM transitions were *m*/*z* 607.2 → 174.1 for CEP and *m*/*z* 237.1 → 194.0 for IS (carbamazepine) with the collision energy of 42 V and 14 V separately. The ESI source conditions were optimized as follows: capillary voltage 0.5 kV, cone voltage 43 V, cone gas 150 L/h, source temperature 150 °C, desolvation temperature 450 °C, and desolvation gas 650 L/h.

#### 4.3.2. Preparation of Stock, Calibration Standards, and Quality Control Samples

The stock solutions (10.0 mg/mL) of CEP and IS were separately prepared in methanol. A battery of working solutions of CEP was diluted with 75% methanol-water. Calibration standards samples were prepared by spiking 5 μL of the working solution with 45 μL of blank plasma to yield a calibration curve with concentrations of 0.5, 1.0, 5.0, 10, 25, 50, and 100 ng/mL, and the QC samples were prepared at the low, medium, and high levels (LQC, MQC, and HQC, respectively) of concentration 1.5, 15, and 75 ng/mL, respectively.

#### 4.3.3. Sample Preparation

The method of protein precipitation was applied for sample preparation, which was very simple and convenient [46,47]. Briefly, 50 μL of the plasma sample was spiked with 150 μL of methanol (containing 10 ng/mL IS), vortex-mixed for 1 min, and then centrifuged at 15,493× *g* for 10 min at 4 °C. Finally, 150 μL of the supernatant was collected for UPLC-MS/MS analysis.

### 4.4. UPLC-MS/MS Method Validation

The method was validated in terms of specificity, linearity, sensitivity, precision, accuracy, matrix, recovery effect, stability, carryover, and dilution effect, in accordance with the international guidelines of the US Food and Drug Administration (FDA) and *Chinese Pharmacopoeia* [48,49].

The selectivity was evaluated by analyzing blank rat plasma samples derived from six independent sources. A 7-point linear calibration curve was constructed using a weighted (1/x^2^) least-squares linear regression by plotting the peak area ratio of analyte/IS versus analyte concentration over the range of 0.5–100 ng/mL. The carryover effect was evaluated by injecting a blank sample after reaching the upper limit of the quantitation sample (ULOQ, 100 ng/mL). The accuracy and precision were determined by analyses of six replicate QC samples at four different concentration levels of 0.5, 1.5, 15, and 75 ng/mL, respectively, in three independent analysis batches on separate days. The accuracy was expressed as a relative error (RE, %), while the intra- and inter-day precision was expressed as the relative standard deviation (RSD, %).

The matrix effect was calculated by the ratio of the peak areas of the post-extraction spiked samples to that of the pure standard solutions at 3 different QC levels in sextuplicate. The extraction recovery was determined by comparing the peak areas of the pre-extraction spiked samples with that of the post-extraction spiked samples at 3 QC levels (*n* = 6). The stability of CEP in the plasma and solution was estimated at 3 QC levels (*n* = 6). Short- and long-term stability studies were performed with the plasma under different storage conditions, specified as follows: 3 h at room temperature (25 °C), 14 h at 4 °C, 8 h in an autosampler at 10 °C, three freezes (−80 °C) and thaw cycles (25 °C), and 30 days at −80 °C. The stability of stock solutions was also assessed after 10-day storage at 4 °C by comparison with the fresh stock. Dilution integrity was evaluated to guarantee the accuracy of quantitation for samples with a concentration above the ULOQ value. Six replicates of the plasma samples containing CEP 5000 ng/mL were prepared and diluted 100-fold with the blank plasma, followed by analyses with freshly established calibration curves.

### 4.5. Preparation of the CEP Solution

We tested different conditions to improve the poor solubility of CEP in water. Ultimately, we found that the acidic vehicle was the key factor for improving CEP dissolution. CEP solutions of concentrations 2.0 and 30 mg/mL were respectively prepared with pH 3.7 and pH 2.8 acidic saline regulated by acetic acid, in which the CEP powder was fully dissolved by vortexing for 20 s. The final pH value of the solutions was detected to be in the range of 5.0–5.5, which could be used for both intravenous and pulmonary administrations in the subsequent animal experiments.

### 4.6. Pharmacokinetics and Bioavailability Studies

To characterize the pharmacokinetics and bioavailability of CEP, 12 SD rats were equally randomized into 3 groups as follows: intravenous, pulmonary, and oral administration at a single dose of 1, 1, and 10 mg/kg, respectively. Intravenous injection of CEP solution (100 μL) was administrated through the lateral tail vein. For the pulmonary drug delivery, the rats were first anesthetized with isoflurane inhalation, and 100 μL of the CEP solution was administrated via the trachea using a nebulizer and laryngoscope; the experiment was performed on a rat surgery board held at the angle of approximately 60° to the horizon. Blood samples (approximately 0.2 mL) were collected from the jugular vein cannulation at 0.017, 0.083, 0.25, 0.5, 1, 2, 4, 7, 12, 24, 36, and 48 h after the drug administration. After centrifugation at 1500× *g* for 10 min, the supernatant plasma was collected and stored at −80 °C until analyses. The pharmacokinetic parameters of CEP were calculated by using a non-compartmental analysis model with the Phoenix WinNonlin software (version 6.4, Pharsight^®^, Cary, NC, USA). The absolute bioavailability (F) of CEP after oral or pulmonary administration was calculated by comparing the respective mean AUC_(0-t)_/dose values with those determined after the intravenous drug administration.

### 4.7. In Vivo Antifibrotic Effect

#### 4.7.1. Experiment Model and Study Group

A total of 30 SD rats, acclimatized for 1 week before the experiment, were randomly assigned to 5 groups, as specified below: (1) control group; (2) BLM-induced pulmonary fibrosis group; (3) BLM-induced pulmonary fibrosis + 5 mg/kg CEP group; (4) BLM-induced pulmonary fibrosis + 15 mg/kg CEP group; (5) BLM-induced pulmonary fibrosis + pirfenidone group (PFD, 100 mg/kg, positive control) [34,50]. All rats, except the control rats, were first anesthetized through isoflurane inhalation and then with 5 mg/kg of BLM via pulmonary administration to induce an inflammatory response and fibrosis. The control group received an equal volume of normal saline. From days 2 to 21, 6 groups of rats received normal saline (control group, pulmonary administration), or CEP solution (BLM + CEP group, pulmonary administration), or pirfenidone (BLM + PFD group, oral administration) for 20 consecutive days (once a day), as illustrated in Table 4. On day 22, the rats were weighed and anesthetized via isoflurane inhalation. Their lung tissues and blood specimens were collected for further analysis.

#### 4.7.2. Measurement of the Lung Index

The lung index analysis is considered to be a simple approach to estimate the extent of lung edema [51]. On the last day, the intact lung tissue was removed, rinsed with pre-cooled saline, dried with an absorbent paper, and then weighed. The lung index was calculated using the following formula:

Lung index = lung weight (mg)/body weight (g)
(1)

#### 4.7.3. Histopathological Examination

To assess the histological markers, the left lung tissues of each rat were fixed in 4% paraformaldehyde for 24 h, then dehydrated, sliced sagittally, and then embedded in paraffin. Then, the lung sections (5 μm thick) were stained with H&E or Masson Trichrome for morphological analysis and the assessment of collagen deposition by light microscopy.

#### 4.7.4. ELISA Assay

ELISA kits were used to determine the contents of cytokines, including IL-6 (abx050126, Abbexa, Cambridge, UK) and TGF-β1 (CZR10-96, Beijing Cheng-zhi-ke-wei Biotechnology Co., Ltd., Beijing, China) in the serum. The operation was performed as per the manufacturer’s instructions.

#### 4.7.5. Western Blotting

The lung tissues were homogenized in RIPA lysis buffer containing protease inhibitor on an ice bath and then centrifuged at 12,000× *g* for 15 min at 4 °C. The supernatant was collected and the protein content was measured with the BCA protein quantitation kit. The samples containing total protein (50 μg) were then separated on 10% SDS-PAGE gel and transferred onto a polyvinylidene difluoride membrane. The membrane was blocked in 5% fat-free milk and incubated with a primary antibody against TGF-β1 (1:5000, Abcam, Cambridge, UK), α-SMA (1:1000, CST, Danvers, MA, USA), and GAPDH (1:5000, CST, USA) overnight at 4 °C. On the next day, the membranes were incubated with horseradish-peroxidase-conjugated secondary antibodies at room temperature for 40 min and then with enhanced chemiluminescence for color development. Image J software was used to analyze the densities of the protein bands. The results were expressed as gray values, and the gray value ratio of the target protein to GAPDH served as the relative level of target protein expression.

#### 4.7.6. Measurement of HYP Content

HYP is a modified amino acid abundant in collagen, whose content characterizes the extent of collagen deposition [41]. The concentration of HYP was determined in the lung tissue homogenates by using the alkaline hydrolysis method (HYP kit, Nanjing Jiancheng Bioengineering Institute, Nanjing, China) as specified by kit manufacturers’ instructions.

### 4.8. Statistical Analysis

Statistical analysis was performed using GraphPad Prism 8.3.0 software, and the values were expressed as mean ± standard deviation (SD). One-way single-factor analysis of variance was used for comparisons among the groups of data, with *p* < 0.05 considered to indicate statistical significance.

## Figures and Tables

**Table 1:** Precision and accuracy of the assay for CEP in rat plasma (*n* = 6).

Analyte	Concentration (ng/mL)		Precision (RSD%)		Accuracy (RE%)
	Nominal	Measured	Intra-Day	Inter-Day	
CEP	0.5	0.532 ± 0.033	12.4	4.9	6.4
	1.5	1.51 ± 0.07	12.4	2.1	0.5
	15	15.2 ± 0.57	6.9	3.1	1.1
	75	72.6 ± 2.3	7.5	2.0	−3.2

**Table 2:** Matrix effects and recovery of CEP in rat plasma (*n* = 6).

Analytes	Concentration (ng/mL)	Matrix (%)		Recovery (%)	
		Mean ± SD	CV (%)	Mean ± SD	CV (%)
CEP	1.5	105.8 ± 1.0	0.9	94.8 ± 4.9	5.2
	15	98.9 ± 1.7	1.7	93.9 ± 3.1	3.3
	75	102.0 ± 2.7	2.7	94.9 ± 1.9	2.0

**Table 3:** Pharmacokinetic parameters of CEP after intravenous (i.v., 1 mg/kg), oral (p.o., 10 mg/kg), and pulmonary administration (p.a., 1 mg/kg) (*n* = 4, mean ± SD).

Parameter	i.v.	Parameter	p.o.	p.a.
C_max_ (ng/mL)	148.8 ± 60.1	C_max_ (ng/mL)	31.8 ± 14.6	65.3 ± 16.1
T_max_ (h)	0.017 ± 0.000	T_max_ (h)	13.50 ± 7.55	0.017 ± 0.000
t_1/2_ (h)	2.80 ± 0.42	t_1/2_ (h)	17.15 ± 3.14	16.35 ± 1.67
Vz (L/kg)	7.01 ± 1.69	Vz/F (L/kg)	218.0 ± 39.8	52.3 ± 9.6
CL (L/h/kg)	1.74 ± 0.33	CL/F (L/h/kg)	9.14 ± 2.92	2.22 ± 0.37
AUC_(0-t)_ (h·ng/mL)	576.2 ± 114.1	AUC_(0-t)_ (h·ng/mL)	757.8 ± 144.7	392.2 ± 43.7
MRT_(0-t)_ (h)	16.0 ± 1.8	MRT_(0-t)_ (h)	20.7 ± 3.5	15.4 ± 1.0
		F (%)	13.15	68.07

C_max_: maximum plasma concentration; T_max_: time to reach C_max_; t_1/2_: elimination half-life; CL: clearance; Vz: apparent volume of distribution; AUC_(0-t)_: area under the plasma concentration-time curve from time zero to the last time point; MRT: mean residence time.

**Table 4:** Scheme of treatment.

Experimental Groups		*n*	Treatment on Day 1	Daily Treatment from Day 2 to Day 21
I	Control	6	Normal saline (p.a.)	Normal saline (p.a.)
II	BLM	6	BLM (p.a. 5 mg/kg)	Normal saline (p.a.)
III	BLM + CEP 5 mg/kg	6	BLM (p.a. 5 mg/kg)	CEP (p.a. 5 mg/kg)
IV	BLM + CEP 15 mg/kg	6	BLM (p.a. 5 mg/kg)	CEP (p.a. 15 mg/kg)
V	BLM + PFD 100 mg/kg	6	BLM (p.a. 5 mg/kg)	PFD (p.o. 100 mg/kg)

p.a.: pulmonary administration; p.o.: oral administration.

## Data Availability

The data presented in this study are available on request from the corresponding author.

## Sample Availability

A sample of the compound cepharanthine is available from the authors.

## References

[1] Wang C., Horby P.W., Hayden F.G., Gao G.F. (2020). A novel coronavirus outbreak of global health concern. Lancet.

[2] Beyerstedt S., Casaro E.B., Rangel É.B. (2021). COVID-19: Angiotensin-converting enzyme 2 (ACE2) expression and tissue susceptibility to SARS-CoV-2 infection. Eur. J. Clin. Microbiol. Infect. Dis..

[3] Thakur S., Sasi S., Pillai S.G., Nag A., Shukla D., Singhal R., Phalke S., Velu G.S.K. (2022). SARS-CoV-2 Mutations and Their Impact on Diagnostics, Therapeutics and Vaccines. Front. Med..

[4] Awadasseid A., Wu Y., Tanaka Y., Zhang W. (2021). SARS-CoV-2 variants evolved during the early stage of the pandemic and effects of mutations on adaptation in Wuhan populations. Int. J. Biol. Sci..

[5] Rehman M.F.u., Akhter S., Batool A.I., Selamoglu Z., Sevindik M., Eman R., Mustaqeem M., Akram M.S., Kanwal F., Lu C. (2021). Effectiveness of Natural Antioxidants against SARS-CoV-2? Insights from the In-Silico World. Antibiotics.

[6] Hossain M.K., Hassanzadeganroudsari M., Apostolopoulos V. (2021). The emergence of new strains of SARS-CoV-2. What does it mean for COVID-19 vaccines?. Expert Rev. Vaccines.

[7] Voysey M., Clemens S.A.C., Madhi S.A., Weckx L.Y., Folegatti P.M., Aley P.K., Angus B., Baillie V.L., Barnabas S.L., Bhorat Q.E. (2021). Safety and efficacy of the ChAdOx1 nCoV-19 vaccine (AZD1222) against SARS-CoV-2: An interim analysis of four randomised controlled trials in Brazil, South Africa, and the UK. Lancet.

[8] Brosh-Nissimov T., Orenbuch-Harroch E., Chowers M., Elbaz M., Nesher L., Stein M., Maor Y., Cohen R., Hussein K., Weinberger M. (2021). BNT162b2 vaccine breakthrough: Clinical characteristics of 152 fully vaccinated hospitalized COVID-19 patients in Israel. Clin. Microbiol. Infect..

[9] Hijikata A., Shionyu-Mitsuyama C., Nakae S., Shionyu M., Ota M., Kanaya S., Hirokawa T., Nakajima S., Watashi K., Shirai T. (2021). Evaluating cepharanthine analogues as natural drugs against SARS-CoV-2. FEBS Open Bio.

[10] Ohashi H., Watashi K., Saso W., Shionoya K., Iwanami S., Hirokawa T., Shirai T., Kanaya S., Ito Y., Kim K.S. (2021). Potential anti-COVID-19 agents, cepharanthine and nelfinavir, and their usage for combination treatment. iScience.

[11] Bailly C. (2019). Cepharanthine: An update of its mode of action, pharmacological properties and medical applications. Phytomedicine.

[12] Chang Y.-K., Huang S.-C., Kao M.-C., Huang C.-J. (2016). Cepharanthine alleviates liver injury in a rodent model of limb ischemia-reperfusion. Acta Anaesthesiol. Taiwanica.

[13] Yamazaki T., Shibuya A., Ishii S., Miura N., Ohtake A., Sasaki N., Araki R., Ota Y., Fujiwara M., Miyajima Y. (2017). High-dose Cepharanthin for pediatric chronic immune thrombocytopenia in Japan. Pediatr. Int..

[14] Kim D.E., Min J.S., Jang M.S., Lee J.Y., Shin Y.S., Park C.M., Song J.H., Kim H.R., Kim S., Jin Y.-H. (2019). Natural Bis-Benzylisoquinoline Alkaloids-Tetrandrine, Fangchinoline, and Cepharanthine, Inhibit Human Coronavirus OC43 Infection of MRC-5 Human Lung Cells. Biomolecules.

[15] Rogosnitzky M., Okediji P., Koman I. (2020). Cepharanthine: A review of the antiviral potential of a Japanese-approved alopecia drug in COVID-19. Pharmacol. Rep..

[16] Zhou Y., Hou Y., Shen J., Huang Y., Martin W., Cheng F. (2020). Network-based drug repurposing for novel coronavirus 2019-nCoV/SARS-CoV-2. Cell Discov..

[17] Sixto-López Y., Correa-Basurto J., Bello M., Landeros-Rivera B., Garzón-Tiznado J.A., Montaño S. (2021). Structural insights into SARS-CoV-2 spike protein and its natural mutants found in Mexican population. Sci. Rep..

[18] Min J.S., Kwon S., Jin Y.-H. (2021). SARS-CoV-2 RdRp Inhibitors Selected from a Cell-Based SARS-CoV-2 RdRp Activity Assay System. Biomedicines.

[19] Ruan Z., Liu C., Guo Y., He Z., Huang X., Jia X., Yang T. (2021). SARS-CoV-2 and SARS-CoV: Virtual screening of potential inhibitors targeting RNA-dependent RNA polymerase activity (NSP12). J. Med. Virol..

[20] Drayman N., DeMarco J.K., Jones K.A., Azizi S.-A., Froggatt H.M., Tan K., Maltseva N.I., Chen S., Nicolaescu V., Dvorkin S. (2021). Masitinib is a broad coronavirus 3CL inhibitor that blocks replication of SARS-CoV-2. Science.

[21] Deng Y., Wu W., Ye S., Wang W., Wang Z. (2017). Determination of cepharanthine in rat plasma by LC-MS/MS and its application to a pharmacokinetic study. Pharm. Biol..

[22] Lu C., Zheng J., Ding Y., Meng Y., Tan F., Gong W., Chu X., Kong X., Gao C. (2021). Cepharanthine loaded nanoparticles coated with macrophage membranes for lung inflammation therapy. Drug Deliv..

[23] Gentile F., Aimo A., Forfori F., Catapano G., Clemente A., Cademartiri F., Emdin M., Giannoni A. (2020). COVID-19 and risk of pulmonary fibrosis: The importance of planning ahead. Eur. J. Prev. Cardiol..

[24] Lechowicz K., Drożdżal S., Machaj F., Rosik J., Szostak B., Zegan-Barańska M., Biernawska J., Dabrowski W., Rotter I., Kotfis K. (2020). COVID-19: The Potential Treatment of Pulmonary Fibrosis Associated with SARS-CoV-2 Infection. J. Clin. Med..

[25] Lu P., Xing Y., Xue Z., Ma Z., Zhang B., Peng H., Zhou Q., Liu H., Liu Z., Li J. (2019). Pharmacokinetics of salvianolic acid B, rosmarinic acid and Danshensu in rat after pulmonary administration of Salvia miltiorrhiza polyphenolic acid solution. Biomed. Chromatogr..

[26] Pierrat P., Wang R., Kereselidze D., Lux M., Didier P., Kichler A., Pons F., Lebeau L. (2015). Efficient in vitro and in vivo pulmonary delivery of nucleic acid by carbon dot-based nanocarriers. Biomaterials.

[27] Marenghi G., Clementino A.R., Fioni A., Buttini F., Sonvico F. (2020). Pulmonary delivery of a p38 α/β; MAP kinase inhibitor: Bioanalytical method validation and biodistribution in rat plasma and respiratory tissues. Eur. J. Pharm. Sci..

[28] Ojo A.S., Balogun S.A., Williams O.T., Ojo O.S. (2020). Pulmonary Fibrosis in COVID-19 Survivors: Predictive Factors and Risk Reduction Strategies. Pulm. Med..

[29] Wei J., Yang H., Lei P., Fan B., Qiu Y., Zeng B., Yu P., Lv J., Jian Y., Wan C. (2020). Analysis of thin-section CT in patients with coronavirus disease (COVID-19) after hospital discharge. J. X-ray Sci. Technol..

[30] Yu M., Liu Y., Xu D., Zhang R., Lan L., Xu H. (2020). Prediction of the Development of Pulmonary Fibrosis Using Serial Thin-Section CT and Clinical Features in Patients Discharged after Treatment for COVID-19 Pneumonia. Korean J. Radiol..

[31] Vasarmidi E., Tsitoura E., Spandidos D.A., Tzanakis N., Antoniou K.M. (2020). Pulmonary fibrosis in the aftermath of the COVID-19 era (Review). Exp. Ther. Med..

[32] Sgalla G., Comes A., Lerede M., Richeldi L. (2021). COVID-related fibrosis: Insights into potential drug targets. Expert Opin. Investig. Drugs.

[33] Bari E., Ferrarotti I., Saracino L., Perteghella S., Torre M., Richeldi L., Corsico A. (2021). Mesenchymal Stromal Cell Secretome for Post-COVID-19 Pulmonary Fibrosis: A New Therapy to Treat the Long-Term Lung Sequelae?. Cells.

[34] George P.M., Wells A.U., Jenkins R.G. (2020). Pulmonary fibrosis and COVID-19: The potential role for antifibrotic therapy. Lancet Respir. Med..

[35] Keum H., Kim D., Kim J., Kim T.W., Whang C.-H., Jung W., Jon S. (2021). A bilirubin-derived nanomedicine attenuates the pathological cascade of pulmonary fibrosis. Biomaterials.

[36] Li X., Liu R., Cui Y., Liang J., Bi Z., Li S., Miao Y., Zhang L., Li X., Zhou H. (2021). Protective Effect of Remdesivir Against Pulmonary Fibrosis in Mice. Front. Pharmacol..

[37] Wu Y., Xu L., Cao G., Min L., Dong T. (2022). Effect and Mechanism of Qingfei Paidu Decoction in the Management of Pulmonary Fibrosis and COVID-19. Am. J. Chin. Med..

[38] Yuan Y., Li Y., Qiao G., Zhou Y., Xu Z., Hill C., Jiang Z., Wang Y. (2021). Hyperbaric Oxygen Ameliorates Bleomycin-Induced Pulmonary Fibrosis in Mice. Front. Mol. Biosci..

[39] Cortijo J., Cerdá-Nicolás M., Serrano A., Bioque G., Estrela J.M., Santangelo F., Esteras A., Llombart-Bosch A., Morcillo E.J. (2001). Attenuation by oral N-acetylcysteine of bleomy-cin-induced lung injury in rats. Eur. Respir. J..

[40] Zhou Y., Li P., Duan J.X., Liu T., Guan X.X., Mei W.X., Liu Y.P., Sun G.Y., Wan L., Zhong W.J. (2017). Aucubin alleviates bleomycin-induced pulmonary fibrosis in a mouse model. Inflammation.

[41] Ali E.N., Mansour S.Z. (2011). Boswellic acids extract attenuates pulmonary fibrosis induced by bleomycin and oxidative stress from gamma irradiation in rats. Chin. Med..

[42] Zhao Y., Yan Z., Liu Y., Zhang Y., Shi J., Li J., Ji F. (2021). Effectivity of mesenchymal stem cells for bleomycin-induced pulmonary fibrosis: A systematic review and implication for clinical application. Stem Cell Res. Ther..

[43] Xu X., Chen P., Wang J., Feng J., Zhou H., Li X., Zhong W., Hao P. (2020). Evolution of the novel coronavirus from the ongoing Wuhan outbreak and modeling of its spike protein for risk of human transmission. Sci. China Life Sci..

[44] You X., Jiang X., Zhang C., Jiang K., Zhao X., Guo T., Zhu X., Bao J., Dou H. (2022). Dihydroartemisinin attenuates pulmonary inflammation and fibrosis in rats by suppressing JAK2/STAT3 signaling. Aging.

[45] Fan H., Wang L.-Q., Liu W.-L., An X.-P., Liu Z.-D., He X.-Q., Song L.-H., Tong Y.-G. (2020). Repurposing of clinically approved drugs for treatment of coronavirus disease 2019 in a 2019-novel coronavirus-related coronavirus model. Chin. Med. J..

[46] Niu L., Li J., Gu R., Wu Z., Zhu X., Gan H., Ma B., Jia B., Wang F., Meng Z. (2018). An optimized LC-MS/MS method for determination of HYNIC-3PRGD2, a new promising imaging agent for tumor targeting, in rat plasma and its application. J. Chromatogr. B.

[47] Dai Y., Tan A.L.C., Chen H., Ong P.S., Xiang X., Wu J., Lin H.-S. (2018). Quantification of desoxyrhapontigenin (4-methoxyresveratrol) in rat plasma by LC-MS/MS: Application to pre-clinical pharmacokinetic study. J. Pharm. Biomed. Anal..

[48] US Food and Drug Administration (FDA) Bioanalytical Method Validation Guidance for Industry. https://www.fda.gov/regulatory-information/search-fda-guidance-documents/bioanalytical-method-validation-guidance-industry.

[49] Pharmacopoeia Commission of P.R.C. (2020). Guidance for Industry: Bioanalytical Method validation. Pharmacopoeia of People’s Republic of China.

[50] Corboz M.R., Zhang J., LaSala D., DiPetrillo K., Li Z., Malinin V., Brower J., Kuehl P.J., Barrett T.E., Perkins W.R. (2018). Therapeutic administration of inhaled INS1009, a treprostinil prodrug formulation, inhibits bleomycin-induced pulmonary fibrosis in rats. Pulm. Pharmacol. Ther..

[51] Mehrzadi S., Hosseini P., Mehrabani M., Siahpoosh A., Goudarzi M., Khalili H., Malayeri A. (2021). Attenuation of Bleomycin-Induced Pulmonary Fibrosis in Wistar Rats by Combination Treatment of Two Natural Phenolic Compounds: Quercetin and Gallic Acid. Nutr. Cancer.

